# Factors influencing survival and short-term outcomes of very low birth weight infants in a tertiary hospital in Johannesburg

**DOI:** 10.3389/fped.2022.930338

**Published:** 2022-09-16

**Authors:** Kristin Ingemyr, Anders Elfvin, Elisabet Hentz, Robin T. Saggers, Daynia E. Ballot

**Affiliations:** ^1^Department of Paediatrics, Institute of Clinical Sciences, University of Gothenburg, Sahlgrenska Academy, Gothenburg, Sweden; ^2^Region Västra Götaland, Department of Paediatrics, The Queen Silvia Children's Hospital, Sahlgrenska University Hospital, Gothenburg, Sweden; ^3^Department of Paediatrics and Child Health, Charlotte Maxeke Johannesburg Academic Hospital, Johannesburg, South Africa; ^4^School of Clinical Medicine, Faculty of Health Sciences, University of the Witwatersrand, Johannesburg, South Africa

**Keywords:** survival, short-term outcomes, infant, prematurity, neonatal mortality, very low birth weight (VLBW), premature neonate, low- and lower-middle-income countries

## Abstract

**Background:**

The neonatal mortality rate in South Africa is lower than the global average, but still approximately five times higher than some European and Scandinavian countries. Prematurity, and its complications, is the main cause (35%) of neonatal deaths.

**Objective:**

To review the maternal, delivery period and infant characteristics in relation to mortality in very low birth weight (VLBW) infants at Charlotte Maxeke Johannesburg Academic Hospital (CMJAH).

**Methods:**

This was a retrospective descriptive study of VLBW infants admitted to CMJAH between 1 January 2017 and 31 December 2018. All infants with a birth weight between 500 to ≤ 1,500 grams were included. The characteristics and survival of these infants were described using univariate analysis.

**Results:**

Overall survival was 66.5%. Provision of antenatal steroids, antenatal care, Cesarean section, female sex, resuscitation at birth, and 5-min Apgar score more than five was related with better survival to discharge. Among respiratory diagnoses, 82.8% were diagnosed with RDS, 70.8% received surfactant therapy and 90.7% received non-invasive respiratory support after resuscitation. At discharge, 59.5% of the mothers were breastfeeding and 30.8% spent time in kangaroo mother care.

**Conclusion:**

The two-thirds survival rate of VLBW infants is similar to those in other developing countries but still remains lower than developed countries. This may be improved with better antenatal care attendance, coverage of antenatal steroids, temperature control after birth, improving infection prevention and control practices, breastfeeding rates and kangaroo mother care. The survival rate was lowest amongst extremely low birth weight (ELBW) infants.

## Introduction

In South Africa, the under-5 mortality rate in 2017 was 37.1 per 1,000 live births (1). South Africa and the rest of Sub-Saharan Africa failed to achieve the fourth Millennium Development Goal from the United Nations of a two-thirds reduction in the under-5 mortality rate (2). This is largely due to the relatively slow decline in the neonatal mortality rate over the last two decades (3).

During the neonatal period the mortality was 10.7 per 1,000 live births (4). While this is lower than the global average during the same time period (18.0 per 1,000 live births), it is higher than rates found in developed countries, such as Japan (0.9 per 1,000 live births), and approximately five times higher than some European and Scandinavian countries (4).

Prematurity, and its complications, is the main cause (35%) of neonatal deaths according to estimates developed by the United Nations Inter-agency Group for Child Mortality Estimation in their report from 2019. The second and third largest reasons contributing to neonatal mortality are intrapartum-related complications (24%) and sepsis (15%) (5). According to a review from The Gambia, factors which contribute to neonatal mortality include lack of antenatal care, birth weight <1,500 grams, hypothermia after birth and delivery outside teaching hospital (6).

The complications of preterm birth are many, due to immaturity of multiple organ systems (7). Some of these are intraventricular hemorrhage (IVH), respiratory distress syndrome (RDS), pulmonary hemorrhage, bronchopulmonary dysplasia (BPD), necrotizing enterocolitis (NEC), patent ductus arteriosus (PDA), and retinopathy of prematurity (ROP).

This study aimed to review the maternal, delivery period and infant characteristics in relation to mortality in VLBW infants at the CMJAH. The acquired information will be useful to improve the neonatal care and neonatal survival at CMJAH.

## Materials and methods

### Study design

This was a retrospective observational study of VLBW infants admitted to CMJAH. All infants with birth weight between 500 to ≤ 1,500 grams, born between 1 January 2017 and 31 December 2018 and admitted to the neonatal unit at CMJAH were included. Infants with incomplete data for birth weight, place of birth and date of outcome were excluded.

#### Setting

Charlotte Maxeke Johannesburg Academic Hospital (CMJAH) is a large tertiary referral hospital within the public hospital framework in South Africa. South Africa is considered a developing country and has limited health resources and high patient numbers. In this setting it is not possible to provide full support to every very low birth weight (VLBW) infant due to necessity of rationing care. Therefore, intermittent positive pressure ventilation (IPPV) after the resuscitation period is not routinely provided to infants who weigh <800 grams in this institution based on anticipated poor outcome, prolonged ventilation, and high use of resources.

At CMJAH there is a quality improvement project called PRINCE—the Project to Improve Neonatal Care—with the purpose to develop targeted interventions to improve the neonatal care and clinical outcome of infants at CMJAH. PRINCE has been accredited as a research programme within the Faculty of Health Science at the University of Witwatersrand.

#### Database and data collection

Data was managed using the Research Electronic Data Capture (REDCap) computer database, hosted by the University of Witwatersrand (8, 9). The REDCap computer database at CMJAH started in 2013 and information is collected at discharge. Medical staff collect the data from the patient records, before it is entered on to a computer summary form and then the database itself, with multiple checks and verification. Records contain information such as demographics, clinical characteristics, hospital course, and outcome at discharge.

#### Variables

Variables were selected from amongst those available from the database and considered as of possible importance for preterm delivery and the short-term outcome. The studied maternal characteristics were: maternal age, multiple gestation, parity, maternal HIV status, maternal syphilis, antenatal care, antenatal steroids. The studied characteristics for the delivery period were: place of birth (inborn or outborn), mode of delivery (vaginal or Cesarean section), 5-min Apgar score. The studied infant characteristics were: gestational age, birth weight, head circumference at birth, sex, birth HIV PCR, initial resuscitation in the delivery room, and hypothermia.

Factors related to hospital stay and neonatal complications that were studied included age on admission, age at outcome, respiratory diagnosis (RDS, congenital pneumonia, pulmonary hemorrhage, pneumothorax), surfactant therapy at any time, respiratory support after initial resuscitation (non-invasive or invasive), any respiratory support after 36 weeks, steroids for BPD, screening for ROP, ROP stage 3 or 4, IVH grade 3 or 4, PDA, NEC grade 2 or 3, early-onset sepsis, late-onset sepsis, surgery for NEC, other surgery, blood transfusion, neonatal jaundice requiring phototherapy, congenital anomalies, Kangaroo Mother Care (KMC), and breastfed at discharge.

### Definitions

VLBW infants were defined as birth weight ≤ 1,500 grams. Extremely low birth weight (ELBW) infants were defined as birth weight ≤ 1,000. Short-term outcome was defined as death or survival to discharge. For diseases, standard definitions as per the Vermont Oxford Network were used (10). Sepsis was defined as blood-culture proven isolation of a pathogenic organism. Early-onset sepsis was defined as onset within 72 h of life and late-onset sepsis after 72 h of life. Patent ductus arteriosus was diagnosed with echocardiography (performed by cardiologists) after clinical suspicion.

Attendance at antenatal care constituted at least one antenatal visit during pregnancy. Place of birth was divided into inborn (infants born within CMJAH) and outborn, including infants born before arrival, at midwife obstetric units or born at other hospitals and referred to CMJAH. Mode of delivery was divided into vaginal delivery (including vertex and breech presentation) and Cesarean section (including elective and emergency cases). Gestational age at birth was decided from the best obstetric estimation available (firstly maternal dates, secondly early ultrasound or thirdly late ultrasound). If none of the above was available, gestational age was estimated using the Ballard Score (11).

Hypothermia was defined as body temperature <36.5 degrees Celsius, measured within 1 h of admission to the neonatal unit. Respiratory support after initial resuscitation was divided into non-invasive and invasive ventilation. Non-invasive respiratory support included nasal-prong oxygen, nasal continuous positive airway pressure (NCPAP) and high flow nasal cannula oxygen, whereas invasive respiratory support included conventional mechanical ventilation and high frequency ventilation. Similarly, respiratory support at 36 weeks was used to determine severity of BPD, based on the level of support of the earlier mentioned alternatives. During the study period, there was a weight cut off for invasive ventilation at 800 grams and for NCPAP at 750 grams. ELBW who did not qualify for invasive or non-invasive ventilation due to their birth weight, were offered surfactant then placed on nasal prong oxygen.

Mothers were screened for HIV at antenatal care visits and again at delivery. Babies born to HIV-positive mothers had an HIV PCR test performed at birth and were put on to HIV prophylaxis dependent on their level of exposure risk. The requirements for surfactant therapy were preterm infants who were hemodynamically stable, with changes suggestive of RDS on chest X-ray, who had respiratory distress and who required fraction of inspired oxygen ≥40% oxygen to keep oxygen saturations >89%. Cranial ultrasound was performed within the first seven days of life and repeated at 10–14 days of life and then again prior to discharge.

All babies with a birth weight ≤ 1,500 grams or gestational age at birth below 32 weeks, were screened for ROP, at 4 to 6 weeks chronological age. Breastfed on discharge included breastmilk only, fortified breastmilk or breastmilk and formula together. KMC included both intermittent and continuous. KMC was introduced once a baby weighed over 1,200 grams, tolerated full enteral feeds, had an adequate weight gain (>15 g/kg/day), was off supplemental oxygen and could maintain temperature and glucose levels. Survival as outcome included discharged to home or transferred to another hospital. Babies were discharged home once they had achieved a weight of 1,600 grams, established enteral feds, were off supplemental oxygen, maintained temperature and glucose levels.

### Statistical analysis

Statistical analysis was performed using IBM SPSS Statistics version 25. Frequencies and percentages were used to describe categorical variables. Continuous variables were described using mean and standard deviation if they were normally distributed, and median and interquartile ranges (IQR) if they were skewed. Univariate analysis was performed to determine significant associations of various factors with survival at discharge. Unpaired *t*-tests were used comparing normally distributed continuous variables and Mann-Whitney *U* tests for skewed distribution. Chi-Square tests were used to compare categorical variables. A *p*-value of <0.05 was considered significant. Only valid cases were analyzed for each variable (i.e., cases with missing data were excluded from the analysis). Thereafter a multiple logistic regression model with mortality as the binary outcome variable was performed. Variables that were (1) significantly associated with mortality in univariate analysis, (2) had a sufficient number of valid cases, (3) passed the assumption of linearity using Box-Tidwell procedure, (4) were not transformed as part of the initial univariate analysis, and (5) were appropriate were included in the model.

### Ethics

Ethical approval for the study was obtained from the Human Research Ethics Committee of the University of the Witwatersrand (clearance certificate number M190874). As this was a retrospective audit of an existing database, informed consent was waived. All methods were carried out in accordance with relevant guidelines and regulations.

## Results

There were 946 VLBW infants admitted during the study period. Eight infants were excluded (seven infants weighed <500 grams at birth, one infant did not have a date of outcome recorded), 938 VLBW infants were included in the study. The overall survival rate was 66.5% (624/938). The mean birth weight was 1,093.6 grams (SD: ± 249.9), mean gestational age at birth was 28.9 weeks (SD: ± 2.8) and median age at outcome was 27.0 days (IQR: 38).

### Maternal details and delivery period

The mean maternal age was 28.8 years (SD: ± 6.4) and 28.5% (222/780) were primiparous. There were 16.6% (152/913) multiple gestation. Risk factors with significant result for mortality related to antenatal care, labor and delivery are presented in Table 1.

**Table 1:** Obstetric risk factors, infant characteristics and prediction of mortality.

**Risk factor (valid cases)**		**Total *n***	**Survived** ***n* (%)**	**Died *n* (%)**	***P* value**	**Odds ratio**	**95% CI of OR**
Sex (938)	Male Female	426 512	260 (61.0) 364 (71.1)	166 (39.0) 148 (28.9)	0.001	1.57	1.20–2.06
Antenatal care (869)	Yes No	687 182	486 (70.7) 91 (50.0)	201 (29.3) 91 (50.0)	<0.001	0.41	0.30–0.58
Antenatal corticosteroids (812)	Yes No	392 420	276 (70.4) 263 (62.6)	116 (29.6) 157 (37.4)	0.021	0.70	0.53–0.94
Mode of delivery (894)	Vaginal Cesarean section	401 493	229 (57.1) 367 (74.4)	172 (42.9) 126 (25.6)	<0.001	0.46	0.34–0.61
Resuscitation at birth (823)	Yes No	427 396	230 (53.9) 316 (79.8)	197 (46.1) 80 (20.2)	<0.001	3.38	2.48–4.62
5-min Apgar Score (810)	≤ 5 > 5	115 695	68 (59.1) 478 (68.8)	47 (40.9) 217 (31.2)	0.042	1.52	1.02–2.28

Valid cases, those with no missing data.

Percentages reported for rows.

CI, Confidence interval.

Among the mothers 29.6% (275/929) were HIV positive and 1.7% (16/868) positive for syphilis. A total of 78.6% (737/938) infants were inborn. Temperature measured within 1 h of admission showed 60.8% (463/762) of the infants had hypothermia. These results were not significant in relation to mortality.

### Infant details

The birthweight, gestational age and head circumference of survivors were significantly greater compared with the infants who died (see Table 2). The 5-min Apgar score of survivors was significantly higher than for the non-survivors (see Table 2). Birth HIV PCR was done in 93.6% (249/266) of cases where the mother was HIV positive and of these 2.8% (7/249) infants were positive for HIV.

**Table 2:** Infant characteristics comparing survivors to non-survivors.

**Category**	**Survivors** ***n* = 624**	**Non-survivors** ***n* = 314**	***P*-value**
Birthweight, g (mean, SD)	1,186.4 (197.8)	909.1 (240.4)	<0.001
Gestational age at birth, weeks (mean, SD)	29.7 (2.5)	27.2 (2.6)	<0.001
Head circumference, cm (mean, SD)	27.8 (2, 2)	25.5 (2.7)	<0.001
5-min Apgar score, (median, IQR)	9 (1)	7 (4)	<0.001

IQR, interquartile range, SD, Standard deviation.

**Figure 1:**
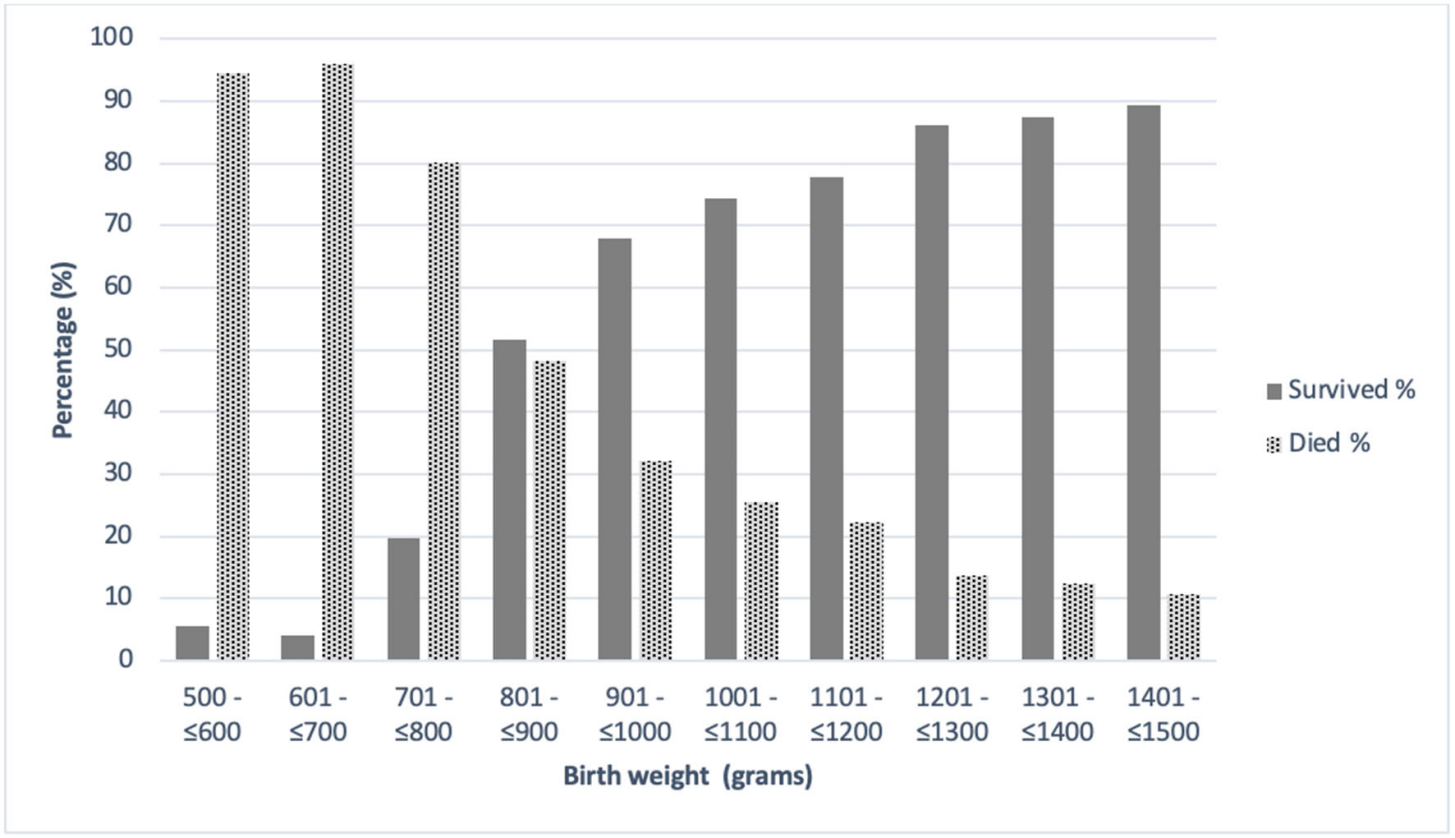
Survival by weight category. Survival of very low birth weight infants by birth weight category.

Survival by birth weight category is shown in Figure 1. The survival of infants with a birthweight from 1,000 to <1,500 grams was 82.2% (514/625) and was significantly greater (*p* < 0.001) compared to the group of extremely low birth weight infants (ELBW) at 35.1% (110/313).

### Hospital stay and neonatal period

Treatments and complications during the neonatal period and hospital stay with statistically significant result for mortality are shown in Table 3.

**Table 3:** Risk factors for mortality related to disease and treatment among studied infants.

**Risk factor (Valid cases)**		**Total *n***	**Survived *n* (%)**	**Died *n* (%)**	***P* value**	**Odds ratio**	**95% CI of OR**
Invasive respiratory support after initial resuscitation (938)	Yes No	245 693	115 (46.9) 509 (73.4)	130 (53.1) 184 (26.6)	<0.001	3.13	2.31–4.23
Respiratory support after 36 weeks (938)	Yes No	148 790	126 (85.1) 498 (63.0)	22 (14.9) 292 (37.0)	<0.001	0.30	0.19–0.48
Surfactant therapy at any time (894)	Yes No	633 261	392 (61.9) 203 (77.8)	241 (38.1) 58 (22.2)	<0.001	2.15	1.54–3.00
Steroids for BPD (823)	Yes No	171 652	159 (93.0) 419 (64.3)	12 (7.0) 233 (35.7)	<0.001	0.14	0.07–0.25
Pulmonary hemorrhage (936)	Yes No	16 920	2 (12.5) 621 (67.5)	14 (87.5) 299 (32.5)	<0.001	14.54	3.28–64.83
Pneumothorax (924)	Yes No	22 902	9 (40.9) 608 (67.4)	13 (59.1) 294 (32.6)	0.012	2.99	1.26–7.07
IVH grade 3 or 4 (610)	Yes No	70 540	19 (27.1) 440 (81.5)	51 (72.9) 100 (18.5)	<0.001	11.81	6.68–20.88
NEC grade 2 or 3 (932)	Yes No	115 817	65 (56.5) 558 (68.3)	50 (43.5) 259 (31.7)	0.015	1.66	1.11–2.47
Surgery for NEC (908)	Yes No	33 875	15 (45.5) 595 (68.0)	18 (54.5) 280 (32.0)	0.008	2.55	1.27–5.13
Other surgery (905)	Yes No	30 875	15 (50.0) 593 (67.8)	15 (50.0) 282 (32.2)	0.049	2.10	1.01-4.36
Neonatal jaundice requiring phototherapy (919)	Yes No	479 440	352 (73.5) 262 (59.5)	127 (26.5) 178 (40.5)	<0.001	0.53	0.40–0.70
Congenital anomalies (925)	Yes No	24 901	8 (33.3) 609 (67.6)	16 (66.7) 292 (32.4)	0.001	4.17	1.77–9.86

Valid cases, those with no missing data.

Percentages are reported for rows.

CI, Confidence interval; BPD, Bronchopulmonary dysplasia; IVH, Intraventricular hemorrhage; NEC, Necrotizing enterocolitis.

There were 90.7% (851/938) of infants who received non-invasive respiratory support after initial resuscitation. Under respiratory diagnosis, 82.8% (777/895) of the infants were diagnosed with RDS and 0.5% (5/935) babies with congenital pneumonia. These results were not significant in relation to mortality. Other respiratory diagnoses which were significant in relation to mortality are included in Table 3.

PDA was reported in 9.2% (85/927) of cases. Among the infants with sepsis there were 4.5% (42/902) with early-onset sepsis and 32% (298/930) with late-onset sepsis. 43.2% (399/924) of the infants received blood transfusion. These results were not significant in relation to mortality.

Screening for ROP occurred in 30.1% (273/906) of the infants and among these 2.9% (8/273) of infants had ROP stage 3 or 4. Breastfeeding at discharge occurred in 59.5% (322/541) of the infants and 30.8% (283/920) spent time in KMC.

There were 22 significant variables on univariate analysis. Six variables (steroids for BPD, pulmonary hemorrhage, pneumothorax, congenital anomalies, IVH grade 3 or 4, and other surgery) were excluded as there were not enough valid cases for both outcomes to include in the logistic regression model. The three continuous variables (birthweight, head circumference, and gestational age) all failed the Box-Tidwell procedure for linearity and were thus excluded from the model. 5-min Apgar score was presented as both an ordinal variable and a transformed categorical variable and was excluded as neither were appropriate for this model. NEC surgery was excluded as it is dependent on NEC 2 or 3, to avoid circular reasoning.

Of the 10 predictor variables included (sex, antenatal care, antenatal steroids, mode of delivery, resuscitation at birth, invasive respiratory support after initial resuscitation, respiratory support after 36 weeks, surfactant at any time, NEC 2 or 3, and neonatal jaundice), seven were statistically significant as shown in Table 4. The logistic regression model explained 44.0% of the variance in outcome and correctly classified 79.5% of cases.

**Table 4:** Multivariate logistic regression for factors associated with mortality in very low birth weight infants at Charlotte Maxeke Johannesburg Academic Hospital.

**Category**	**OR**	**95% CI**	***P*-value**
Invasive ventilation	7.314	4.373–12.232	<0.001
Resus at birth	3.144	2.091–4.726	<0.001
Surfactant therapy	2.329	1.447–3.747	<0.001
Antenatal care	0.410	0.253–0.666	<0.001
Mode of delivery	0.311	0.202–0.480	<0.001
NNJ requiring phototherapy	0.291	0.194–0.438	<0.001
Respiratory support at 36 weeks	0.033	0.014–0.079	<0.001

CI, confidence interval; NEC, necrotizing enterocolitis; OR, odds ratio.

## Discussion

This retrospective review showed an overall two-thirds survival of VLBW infants. This is decreased when compared with the results from previous studies at the same unit where the overall survival of VLBW infants in 2006/2007 was 70.5% and in 2013 was 73.4% (12, 13). These survival rates are similar to those found in other parts of South Africa namely the Eastern Cape province (68.0%), but lower than reported in the Western Cape (81.7%) and in Limpopo (77.4%) (14–16). Further afield, the survival rate in this study compares to other developing countries (like India and Iran) (17, 18) but still remains lower than developed countries (19).

The survival of ELBW in this study is comparable to that found in the 2006/2007 study (13), but lower than the more recent study from the same unit in 2013 (12). Sadly, our results amount to only about half the survival rate that was achieved in a study from the Western Cape (20).

A possible reason for the lower survival rate could be the increase in late-onset sepsis in this study. In 2013, 19% of the VLBW infants at CMJAH had late-onset sepsis, whereas in this study there was an incidence of 32% (12). This may be a consequence of over-crowding or poor adherence to infection prevention and control practices.

In this study 78.6% of the infants were inborn which could be compared with 81.5% in 2006/2007 and 84.3% in 2013 (12, 13). The result was not significant in relation to mortality, which it was in 2006/2007 and 2013. A study in Cape Town, South Africa, found that being inborn was significantly associated with improved survival and decreased morbidity: mothers of inborn infants were more likely to receive antenatal care and antenatal steroids, inborn infants required less ventilatory support, surfactant administration and developed less late-onset sepsis, IVH and BPD (21).

When it comes to antenatal steroids, the coverage in this study had improved slightly (from 39.1 to 48%) compared to 2013 (12). However, this remains poor compared to other South African studies (15, 16, 22). Despite this improvement the goal should be even higher since antenatal steroids have an effect on RDS (hence the amount of respiratory support required for patients), as well as the rates of NEC and IVH (23). Lategan et al. found that exposure to any antenatal steroids was associated with a nearly three-quarter reduction in mortality in their cohort of preterm infants ≤ 1,800 (15). Concerted efforts need to be made to further improve the coverage of antenatal steroids.

Despite the modest improvement in antenatal steroid coverage, the rates of NEC (12.3% vs. 7.3%) and IVH (11.5% vs. 7.9%) in this study has increased compared to 2013 (12). The increased rate of NEC may be due to the increased rate of late-onset sepsis as mentioned above, and vice versa. The increased rate of IVH may be due to increased screening.

When measuring body temperature within 1 h of admission, 60.8% of the infants had hypothermia (skin temperature <36.5 degrees Celsius). Earlier studies done at the same department had not stated their definition of hypothermia, so no comparison could be made. However, this is interpreted as a large number and needs to be improved. Hypothermia increases morbidity (of which RDS is one) and mortality (22). This may have contributed to the large number of infants diagnosed with RDS in this study. Simple, cost-effective measures can be employed to prevent hypothermia, such as increasing awareness of its dangers, regular body temperature monitoring, warmed delivery rooms, providing infants with caps and plastic bags to limit heat loss, and making use or servo-controlled incubators and radiant warmers (which are already in use).

Several diseases showed significant association with mortality, such as pulmonary hemorrhage, congenital anomalies, IVH and NEC. This indicates the severity of these conditions. In addition, surgery for NEC and other surgery were also significantly associated with mortality, although that may be related to the severity of the condition rather than the surgery itself. Predictably, infants with congenital anomalies had higher mortality. It is unit policy not to offer invasive ventilation to infants with congenital anomalies with poor prognosis (e.g., Trisomy 13 and 18).

Taking logistic regression into consideration, receiving any respiratory support during initial resuscitation, surfactant at any time, as well as invasive ventilation after initial resuscitation was associated with higher odds of mortality. The high use of surfactant replacement therapy found in this study is concerning. During the study period, delays were frequently encountered when instituting NCPAP due to equipment shortages. This may have caused infants to deteriorate sufficiently to necessitate surfactant replacement therapy. Efforts have been made to address the delays and improve the use of NCPAP, thereby decreasing the use of surfactant therapy.

Several improvements can be made in the neonatal care prior to discharge. For instance, screening for ROP is supposed to be done on all VLBW infants and was only performed in one third of them prior to discharge in this study. This is still an improvement compared to 2006/2007 when 18.3% of the infants were screened for ROP prior discharge (13). Since the infants are discharged at 1,600 grams, the appropriate time for screening may be after discharge—infants were referred for ophthalmological screening as an outpatient.

In recent years, efforts have been made to increase the number of breastfed infants at discharge, considering the well-documented benefits of breastfeeding and its affordability. To this end, several lactation consultants have been employed. As a result, the number of breastfed infants at discharge has nearly doubled from 2013 (30.5%) to the current rate in this study, which is commendable (12).

On the other hand, KMC has decreased in this study compared with 2006/2007 (30.8% vs. 44.5%) (13). The limitations of KMC in this unit is the availability of mothers—many are single parent households and have to be at home looking after other children. It is important to improve these numbers again since KMC has shown positive results when it comes to better weight gain, earlier hospital discharge and higher exclusive breastfeeding rates in VLBW infants (24, 25).

### Limitations

We acknowledge several limitations to this study. This was a retrospective analysis of an existing database, so some information was missing. Additionally, certain data such as usage of parental nutrition and antibiotics usage, is not routinely collected in the database and were therefore not analyzed. Our study favored maternal dates and Ballard score to determine gestational age as access to early ultrasound is poor. The short-term outcome of survival to discharge included infants discharged home and to other hospitals. We did not look at categorizing the timing of when deaths took place which would be useful in identifying trends. Importantly, this is not a population-based study. This study was conducted in a referral hospital with access to the appropriate level of care, as per national guidelines.

## Conclusion

This study showed a two thirds survival rate in VLBW infants, which is similar to those in other developing countries (like India and Iran) but still remain lower than developed countries. A large number of patients are placing a burden on a hospital with limited resources. The survival rate can be improved by increasing antenatal care attendance, coverage of antenatal steroids, improving temperature control to decrease the high rates of hypothermia, improving infection prevention and control practices, breastfeeding rates and the use of KMC. Improved screening for morbidities such as ROP and IVH should also be emphasized.

## Data availability statement

The raw data supporting the conclusions of this article will be made available by the authors, without undue reservation.

## Ethics statement

The studies involving human participants were reviewed and approved by University of the Witwatersrand Human Research Ethics Committee. Written informed consent from the participants' legal guardian/next of kin was not required to participate in this study in accordance with the national legislation and the institutional requirements.

## Author contributions

KI conceptualized and designed the study, collected data, carried out data analysis, drafted the initial manuscript, revised the manuscript, and approved the final manuscript. AE, EH, and RS participated in protocol development, supervised the study, reviewed and revised the manuscript, and approved the final manuscript. DB supervised the study, revised the manuscript, and approved the final manuscript. All authors contributed to the article and approved the submitted version.

## Funding

This study was financially supported by Stena A. Olssons Stiftelse för Forskning och Kultur and Adlerbertska Scholarship Foundation with their scholarships. Further, this work was supported by grants from the Swedish state under the agreement between the Swedish government and the county councils, the ALF agreement (ALFGBG- 117661).

## Conflict of interest

The authors declare that the research was conducted in the absence of any commercial or financial relationships that could be construed as a potential conflict of interest.

## Publisher's note

All claims expressed in this article are solely those of the authors and do not necessarily represent those of their affiliated organizations, or those of the publisher, the editors and the reviewers. Any product that may be evaluated in this article, or claim that may be made by its manufacturer, is not guaranteed or endorsed by the publisher.

## Abbreviations

BPD: Bronchopulmonary dysplasia
CMJAH: Charlotte Maxeke Johannesburg Academic Hospital
ELBW: Extremely low birth weight
IPPV: Intermittent positive pressure ventilation
IVH: Intraventricular hemorrhage
KMC: Kangaroo Mother Care
NCPAP: Nasal continuous positive airway pressure
NEC: Necrotizing enterocolitis
NNJ: Neonatal jaundice
PDA: Patent ductus arteriosus
PRINCE: Project to Improve Neonatal Care
RDS: Respiratory distress syndrome
REDCap: Research Electronic Data Capture
ROP: Retinopathy of prematurity
VLBW: Very low birth weight.
